# Dysmaturation of Somatostatin Interneurons Following Umbilical Cord Occlusion in Preterm Fetal Sheep

**DOI:** 10.3389/fphys.2019.00563

**Published:** 2019-05-22

**Authors:** Maryam Ardalan, Pernilla Svedin, Ana A. Baburamani, Veena G. Supramaniam, Joakim Ek, Henrik Hagberg, Carina Mallard

**Affiliations:** ^1^Centre for Perinatal Medicine and Health, Institute of Neuroscience and Physiology, Sahlgrenska Academy, University of Gothenburg, Gothenburg, Sweden; ^2^Centre for the Developing Brain, Department of Perinatal Imaging and Health, School of Biomedical Engineering and Imaging Sciences, King’s College London, London, United Kingdom; ^3^Centre for Perinatal Medicine and Health, Institute of Clinical Sciences, Sahlgrenska Academy, University of Gothenburg, Gothenburg, Sweden

**Keywords:** GABA, interneurons, somatostatin, stereology, preterm

## Abstract

**Introduction:**

Cerebral white matter injury is the most common neuropathology observed in preterm infants. However, there is increasing evidence that gray matter development also contributes to neurodevelopmental abnormalities. Fetal cerebral ischemia can lead to both neuronal and non-neuronal structural-functional abnormalities, but less is known about the specific effects on interneurons.

**Objective:**

In this study we used a well-established animal model of fetal asphyxia in preterm fetal sheep to study neuropathological outcome. We used comprehensive stereological methods to investigate the total number of oligodendrocytes, neurons and somatostatin (STT) positive interneurons as well as 3D morphological analysis of STT cells 14 days following umbilical cord occlusion (UCO) in fetal sheep.

**Materials and Methods:**

Induction of asphyxia was performed by 25 min of complete UCO in five preterm fetal sheep (98–100 days gestational age). Seven, non-occluded twins served as controls. Quantification of the number of neurons (NeuN), STT interneurons and oligodendrocytes (Olig2, CNPase) was performed on fetal brain regions by applying optical fractionator method. A 3D morphological analysis of STT interneurons was performed using IMARIS software.

**Results:**

The number of Olig2, NeuN, and STT positive cells were reduced in IGWM, caudate and putamen in UCO animals compared to controls. There were also fewer STT interneurons in the ventral part of the hippocampus, the subiculum and the entorhinal cortex in UCO group, while other parts of cortex were virtually unaffected (*p* > 0.05). Morphologically, STT positive interneurons showed a markedly immature structure, with shorter dendritic length and fewer dendritic branches in cortex, caudate, putamen, and subiculum in the UCO group compared with control group (*p* < 0.05).

**Conclusion:**

The significant reduction in the total number of neurons and oligodendrocytes in several brain regions confirm previous studies showing susceptibility of both neuronal and non-neuronal cells following fetal asphyxia. However, in the cerebral cortex significant dysmaturation of STT positive neurons occurred in the absence of cell loss. This suggests an abnormal maturation pattern of GABAergic interneurons in the cerebral cortex, which might contribute to neurodevelopmental impairment in preterm infants and could implicate a novel target for neuroprotective therapies.

## Introduction

Preterm birth and its associated complications are among the most serious global health issues that modern society faces (Blencowe et al., 2013). Particularly, extreme prematurity (<28 weeks gestation) is associated with poor neurodevelopmental outcome with increased prevalence of cognitive and motor delays (Pascal et al., 2018). The etiology of preterm brain injury is likely to be multifactorial, but circulatory disturbances and inflammation are the critical contributing factors in the pathophysiology of impairment of brain development (Hagberg et al., 2015). Neurodevelopmental disability in infants and children born preterm, so called encephalopathy of prematurity, is associated with impaired cerebral maturation, including white and gray matter volumes, cortical folding, and gyral complexity (Boardman et al., 2006; Volpe, 2009). There is also evidence of delayed cellular maturation, reduced dendritic arborization, impaired synaptogenesis, and connectivity (Tau and Peterson, 2010; Ball et al., 2013), but limited knowledge on specific cell populations.

Depending on the brain region, GABAergic cortical interneurons represent about 10–20% of the neurons within the neocortex (Le Magueresse and Monyer, 2013). These cells control the excitation/inhibition balance, which is crucial for normal brain development and cortical plasticity (Scheyltjens and Arckens, 2016; Fowke et al., 2018). Thus, injury to the immature brain has the potential to affect GABAergic circuitry and cortical function with the consequence of several neurologic disorders (Powell et al., 2003; Butt et al., 2017). Somatostatin (STT)-positive GABAergic neurons are one of the most prevalent populations of early born interneurons with a crucial role in early cortical circuit formation (Rudy et al., 2011). STT is not only a marker of specific types of interneuron but also an inhibitory neuropeptide released from GABAergic neurons (Ludwig and Pittman, 2003; Yavorska and Wehr, 2016). Cortical interneuron subtypes are specified during the fetal period, followed by migration and then differentiation when reaching their cortical destination (Wonders and Anderson, 2006). The timing of migration of STT progenitor cells indicate that they are the first interneurons that migrate to deep layers of the cortical plate during brain development (Miyoshi and Fishell, 2011). Early-born STT neurons localize mainly in cortical layer 5/6 and they persist throughout development in deep layers of the cortex (Rudy et al., 2011). Due to their special characteristics and connectivity, STT interneurons regulate brain plasticity by mediating the maturation of deep layer cortical circuits (Liguz-Lecznar et al., 2016; Tuncdemir et al., 2016). Experimental evidence in neonatal rats indicates that perinatal asphyxia can cause motor deficits related to the loss of GABAergic neurons including calbindin- and parvalbumin-positive interneurons in striatum (Van de Berg et al., 2003). A recent study showed that parvalbumin-positive neurons are reduced following cerebral ischemia in late gestation fetal sheep (Fowke et al., 2018). Post-mortem studies in preterm infants born at 25–32 weeks gestation, demonstrate that in addition to oligodendrocyte loss and axonal disruption, the number of GABAergic interneurons is significantly decreased in brains with white matter lesions (Robinson et al., 2006). More importantly, disruption of the early (but not late) STT interneuron network resulted in impairment of synaptic maturation of thalamocortical inputs onto parvalbumin interneurons (Tuncdemir et al., 2016). However, the effect of perinatal asphyxia at mid-gestation on number and maturation of STT interneurons is not known. In this study we aimed to examine the morphology and distribution of pathological changes of STT-positive GABAergic interneurons following transient *in utero* asphyxia in fetal sheep. *In utero* asphyxia in preterm fetal sheep is a suitable animal model to study complex pathophysiological processes that contribute to brain injury in the preterm infant (Bennet et al., 2012). Specifically, with respect to neuropathology, sheep have similar proportions of gray and white matter as the human (Mallard et al., 2003; Koehler et al., 2018). In these studies, we used a well-established animal model in preterm fetal sheep where asphyxia is induced by transient umbilical cord occlusion (UCO) at midgestation, which is equivalent to 25–30 weeks gestation in the human with respect to brain development (Mallard et al., 1994).

## Materials and Methods

### Fetal Surgery and Umbilical Cord Occlusion

Animal experiments were approved by the local Animal Ethics Committee of Gothenburg (No. 166/13) and performed according to the guidelines for animal experimentation by the Swedish Department of Agriculture. Eight time-mated pregnant ewes were fasted overnight and then underwent aseptic surgery at 95–96 days gestation as previously described (Mallard et al., 2003). Prior to anesthesia induction, the ewe was given Stesolid (Diazepam, 0.1–0.2 mg/kg, i.v.). Anesthesia was induced by sodium pentothal (13 mg/kg, i.v.), followed by intubation and isoflurane (1.5%) and the ewe was also given one injection of Temgesic (Buprenorphine, 0.005–0.02 mg/kg, i.v.) and Garamycin (Gentamicin, 5 mg/kg, i.m). The uterine horn was exposed through a midline incision and a small hysterectomy incision was made over the fetal head through the uterine wall, parallel to any vessels. An inflatable silastic cuff was placed around the umbilical cord (OCHD16, DocXS Biomedical Products, Ukiah, CA, United States). Polyvinyl catheters (i.d. 1 mm, Smiths Medical and tip 0.4 mm, Agnthos, Sweden) were inserted into each brachial/axillary artery and brachial vein. An amnion catheter (i.d. 2.0 mm, Portex, Smiths Medical, Minneapolis, MN, United States) was secured to the ear. In case of twins, only one fetus was instrumented. At the end of the operation, catheters were filled with 50 IU/ml heparinised saline. The uterus was closed in two layers and catheters exteriorized via a trocar. One catheter was placed in the tarsal vein of the ewe. Sheep were allowed to recover for 3–5 days following surgery before experiments began. During this period Gentaject (Gentamicin; 5 mg/kg, i.v.) was administered to the ewe daily.

Induction of asphyxia was performed by 25 min of complete UCO at 98–100 days gestation as previously described (Mallard et al., 2003).

### Tissue Processing and Immunohistochemistry

**FIGURE 1:**
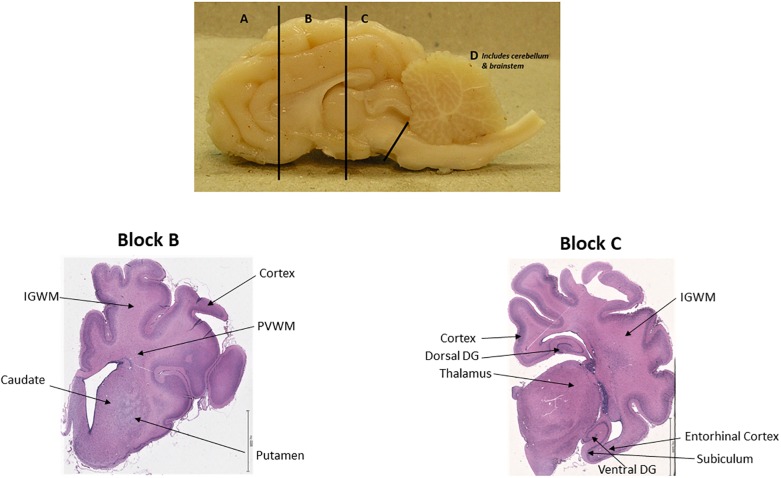
One hemisphere of sheep brain that is cut into four blocks (top); Brain regions of interest in block B and block C. Scale bar = 6.5 mm.

A total of five fetuses with UCO and seven non-occluded twins were included in the study. Ewes were euthanized by a maternal intravenous injection of sodium pentobarbitone 2 weeks after UCO (at 112–114 days gestation). The fetuses were immediately removed, perfusion fixed *in situ* through the carotid arteries with saline (0.9%) followed by 4% paraformaldehyde (PFA) and then brains were removed and further immersion-fixed in 4% PFA until processing (at least 1 month). Brains were separated into left and right hemisphere. The right hemisphere was cut into four coronal blocks (A–D) at a thickness of 5 mm (Figure 1), blocks were separately paraffin embedded and then sectioned on a microtome (Thermo Scientific^TM^ HM 355S Automatic Microtome) based on a systematic sampling principle. Following deparaffinization and rehydration, immunohistochemistry was carried out on paraffin sections by boiling in citrate buffer for antigen retrieval and blocking for endogenous peroxidase (3% H_2_O_2_ in PBS) for 10 min. Non-specific binding was blocked by 4% serum for 30 min in room temperature, followed by incubation with primary antibodies to quantify the number of neurons [monoclonal mouse anti-NeuN (Millipore MAB377) 1:250]; transcription factor expressed in all oligodendrocytes throughout their lineage (1:100 poly-clonal rabbit anti-Olig2, Chemicon, AB9610); immature and mature oligodendrocytes (CNPase, monoclonal mouse, 1:200; Sigma-Aldrich, C5922) and STT (1:100 monoclonal rat anti-STT, IgG2b, Clone YC7, Abcam ab150348) in PBS overnight at 4°C. The next day, sections were incubated with appropriate secondary antibodies [Horse-anti-mouse biotinylated, Goat-anti-rabbit biotinylated, Goat-anti-rat biotinylated (Vector)] (1:250) in PBS for 1 h in room temperature, followed by addition of ABC solution [VECTASTAIN Elite ABC HRP Kit (Peroxidase, Standard, PK-6100)] for 1 h. Visualization of stained cells was performed by 3,3′-diaminobenzidine (DAB) for 10 min, after which slides were coverslipped.

### Estimation of the Total Number of Cells in Brain Regions

Unbiased estimation of the total number of neurons, oligodendrocytes (mature and immature), and STT interneurons was performed by applying the optical fractionator method (Gundersen, 1986). The newCAST software (Visiopharm, Hørsholm, Denmark) was used with a light microscope (Leica DM6000 B, Germany) modified for stereology with a digital camera (Leica DFC 295, Germany) and a motorized microscope stage (Ludl MAC 5000, United States) and 63X oil-immersed lens. Cells were analyzed once the soma of the cells were in focus and inside the unbiased counting frame. For analysis, a 10-μm height disector was applied to each section. Delineation of the area of interest was done using a 5× objective lens. The calculation for counting the number of cells was done according to the applied method in Ardalan et al. (2016) paper.

$$N=\frac{1}{\mathrm{SSF}}\cdot \frac{1}{\mathrm{ASF}}\cdot \frac{1}{\mathrm{HSF}}\cdot \Sigma Q^{-}$$

*N* is the total number of cells per brain region; Σ*Q*^−^ is the number of counted cells; *SSF* is the section sampling fraction; *ASF* is an area sampling fraction; and *HSF* is the height sampling fraction (1/10).

### Acquisition of Images and Morphological Analysis of Somatostatin Interneurons

A systematic set of Z-stacks of the brain regions including cortex, putamen, caudate, subiculum, and entorhinal cortex (EC) on the STT stained sections were captured by using a 63X oil-immersed lens on a light microscope modified for stereology. Application of this method of image acquisition, made it possible to Z-stack capture of more than one interneuron in one image. The height of the Z stacks was 16 μm equal to the thickness of sections, and the acquisition of images was performed in steps of 1 μm.

The captured images were analyzed by using Filament Tracers algorithm in Imaris software (Version 8.4, Bitplane A.G., Zurich, Switzerland), and the morphological parameters of STT interneurons including the number of dendrites, the total length of the dendrites, and the sholl intersections were quantified. Sholl analysis was performed to explore the complexity of cellular branches (Anzabi et al., 2018). By selecting the center of STT soma as a reference point, the length of the dendrites was measured based on the radial distance from the reference point (soma) in 20 μm. The sum of the number of branching intersections for all circles, for each cell, and the mean of the results from 15 STT cells per region per animal was quantified. The reliability of the STT reconstruction was considered by following sampling/selection criteria; (1) the STT cell soma had to be in the middle of the section thickness with the clear border; (2) the STT cell had to have intact clear dendrites; and (3) the dendrites of the selected cell should not be obscured with the branches of nearby cells or the background staining.

All measurements were performed by a researcher blinded to the groups of animals.

### Neuropathology Assessment

Two anatomical levels of the brain at the level of lateral ventricle/basal ganglia and at the level of the thalamus and hippocampus, corresponding to section 720 and section 1120, respectively, in the Sheep Ovis Aries atlas^1^ were used for analysis (Figure 1). Brain sections was deparaffinised and stained with acid fuchsin/thionin for structural analysis (Bennet et al., 2012). Briefly, sections were stained with thionin for 4 min, followed with rinsing in distilled water, acid fuchsin staining for 20 s and dehydrated through a graded series of alcohol, cleared in xylene and coverslipped.

Acid-fuchsin/thionin-stained sections were examined for gross structural damage, including areas of pallor, neuronal eosinophilia, karyopyknosis, cavitation, necrosis, and infarction. The cortex, subcortical and periventricular white matter (PVWM), striatum/basal ganglia and hippocampus were all assessed. A neuropathology score was assigned to each brain region and section according to criteria described in Table 1. All neuropathology scoring was performed by an assessor blinded to the identity of the animal group.

**Table 1:** Scoring template for neuropathology based upon tissue damage in acid-fuchsin/thionin stained sections.

Neuropathology scoring	
**Acid-fuschsin/thionin**	
0	Absent
1	Neuronal eosinophilia, karyopyknosis; small patches of necrotic cells, small areas of pallor
2	Areas with increased cellularity; moderate patches of necrotic cells, moderate areas of pallor
3	PVL/IVH; severe tissue loss; large infarcts/cavitation

*Adapted from Fraser et al. (2007) and Dean et al. (2011). PVL, periventricular leukomalacia; IVH, intraventricular hemorrhage.*

### Statistical Analysis

All data was analyzed by using IBM Corp. Released 2013 (IBM SPSS Statistics for Windows, Version 22.0. IBM Corp., Armonk, NY, United States) and graphs were created by using Sigmaplot 12.5 (SYSTAT, San Jose, CA, United States). Prior to statistical tests, normal distribution of data was tested by making a Q–Q plot of the data. The variance homogeneity of data was tested by Levene’s test. In the cases that the distribution of data was not normal and/or data variance was different, a logarithmic transformation was performed before statistical testing was employed. The collected data from the two groups (UCO and control) was compared using the independent *t*-test. In all cases, the significance level was set at *p* < 0.05. Data are presented as mean ± SD.

## Results

### Gross Neuropathology

There were no large areas of infarcts or PVL/IVH in any of the UCO animals, however, 3/5 animals displayed areas of pallor and small patches of necrotic cells in the subcortical and PVWM (Table 2). There was no visible neuropathological abnormality, based on the gross neuropathological score, in the cortex, basal ganglia, and hippocampus (Table 2).

**Table 2:** Descriptive information of neuropathological scores in different brain regions for each UCO animal.

	Sheep # 4A	Sheep # 5A	Sheep # 12A	Sheep # 14	Sheep # 15A
	
	AF/T^∗^ score	AF/T score	AF/T score	AF/T score	AF/T score
Periventricular white matter	1	1	1	0	0
Subcortical white matter	2	2	2	0	0
Cortex/gray matter	0	0	0	0	0
Basal ganglia/striatum	0	0	0	0	0
Hippocampus	0	0	0	0	0

*^∗^AF/T, acid-fuchsin/thionin.*

### Effect of UCO on Number of STT Interneurons

**FIGURE 2:**
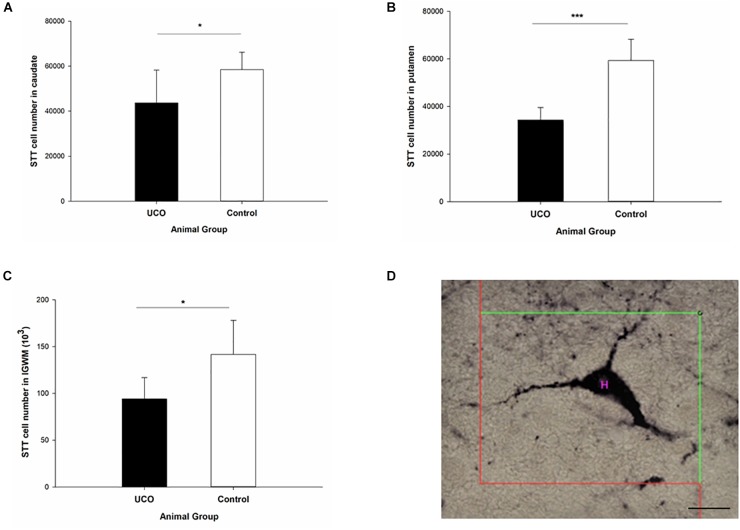
The effect of umbilical cord occlusion (UCO) on the total number of somatostatin (STT) interneurons in block B regions of interest: **(A)** caudate; **(B)** putamen; **(C)** intragyral white matter (IGWM) (^∗^*p* < 0.05, ^∗∗∗^*p* < 0.001). **(D)** Illustration of counting the number of STT interneuron inside the unbiased counting frame on the light microscope connected to newCAST software with a 63X oil immersion lens. Mean ± SD. Scale bar = 15 μm.

**FIGURE 3:**
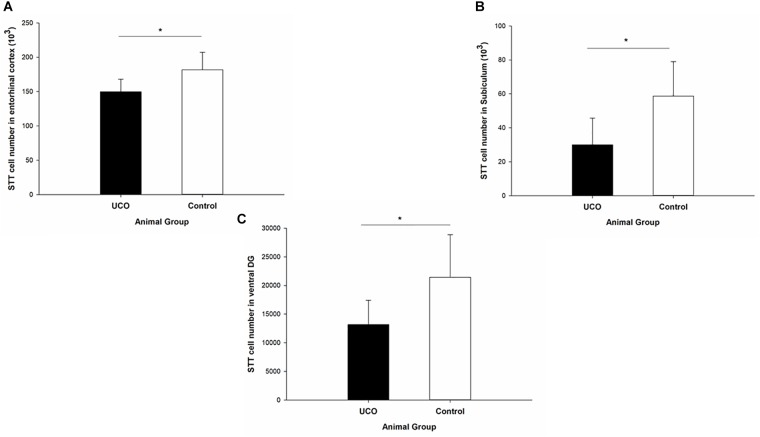
The effect of UCO on the total number of STT interneurons in block C regions of interest: **(A)** entorhinal cortex, **(B)** subiculum, and **(C)** ventral DG. Mean ± SD. ^∗^*p* < 0.05. DG, dentate gyrus.

The number of STT interneurons was counted in different brain regions in block B: cortex, intragyral white matter (IGWM), PVWM, caudate and putamen (Figure 2) and block C: EC, subiculum, and dentate gyrus (DG) subregion of dorsal and ventral hippocampi (Figure 3). The UCO group showed a significant reduction in the number of STT positive interneurons in the caudate, putamen and IGWM compared to the control group (*p* = 0.04; *p* = 0.000; *p* = 0.030; Figure 2 and Supplementary Table 1). In the DG area of hippocampus, a significant effect of UCO was observed on the number of STT interneurons in the ventral part of the DG compared with control group (*p* = 0.05), however, there was no significant change in the number of STT cells in the dorsal DG in UCO group (*p* = 0.21). Moreover, our results indicated significant reduction in the number of STT cells in the subiculum of UCO animals compared to the control group (*p* = 0.02). The number of STT-positive interneurons in the EC was significantly lower in UCO group vs. control group (*p* = 0.03; Figure 3 and Supplementary Table 1), while the other parts of cortex, were virtually unaffected by the UCO (*p* > 0.05).

### Effect of UCO on the STT Interneuron Morphology

**FIGURE 4:**
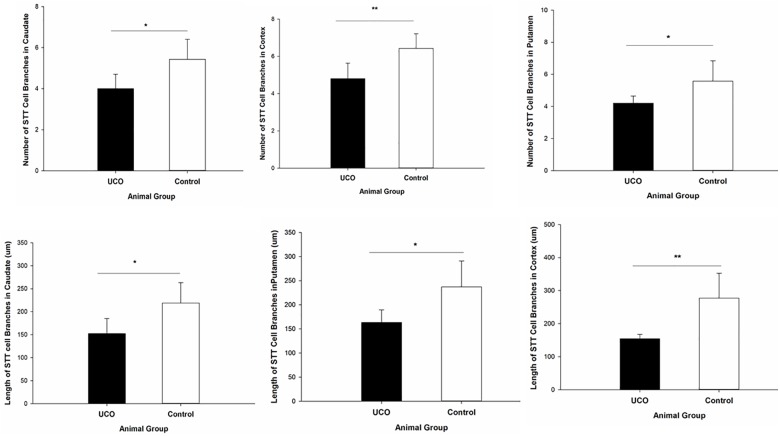
Morphological analysis of STT interneurons showed a significant effect of UCO on the length and number of the STT dendrites in cortex, caudate, and putamen. Mean ± SD. ^∗^*p* < 0.05, ^∗∗^*p* < 0.01.

**FIGURE 5:**
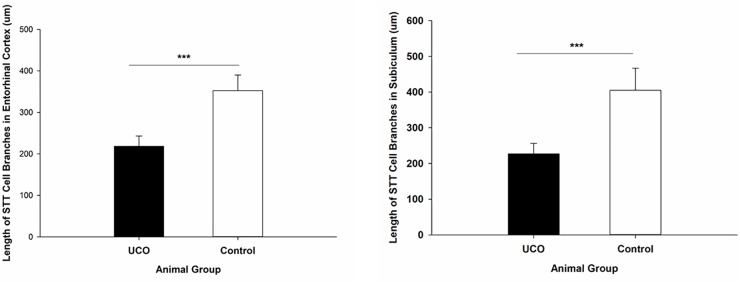
Morphological analysis of STT interneurons showed a significant effect of UCO on the length of the STT dendrites in subiculum and EC. Mean ± SD. ^∗∗∗^*p* < 0.001.

The morphological analysis of STT positive cells showed that the total length of dendrites was significantly shorter in layer six of the cortex, caudate, and putamen in UCO group compared to control group, respectively (*p* = 0.005; *p* = 0.01; *p* = 0.01) (Figure 4). Moreover, UCO resulted in shorter length of STT dendrites in subiculum and EC (*p* < 0.001; *p* < 0.001) (Figure 5).

**FIGURE 6:**
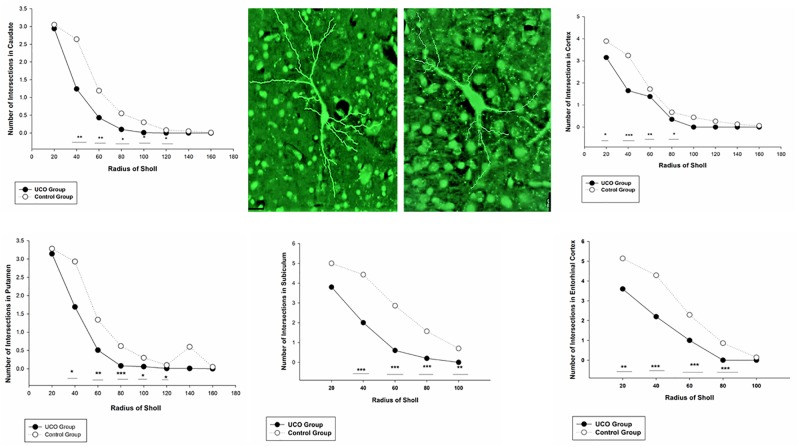
Branching pattern alterations of STT interneurons in cortex, caudate, putamen, subiculum, and EC using Sholl analysis in UCO and control groups. The number of branching intersections at various distances away from the cell soma was significantly lower in the UCO group vs. control group. ^∗^*p* < 0.05, ^∗∗^*p* < 0.01, ^∗∗∗^*p* < 0.001. Example of morphological analysis of STT interneuron in the putamen area of brain from control (left) and UCO (right) animals. Scale bar = 20 μm.

The number of dendrites of STT cells in the cortex, caudate, and putamen was remarkably higher in control group in comparison with UCO group (*p* = 0.006; *p* = 0.02; *p* = 0.04) (Figure 4). The number of STT dendrites in subiculum was significantly lower in UCO group compared with control group (*p* = 0.002), while there was no significant change in numbers of STT dendrites in the EC following UCO compared to control group (*p* = 0.38) (Figure 5). Regarding the effect of UCO on the complexity of STT dendrites in brain regions, sholl analysis showed significant reduction of the STT arborization in cortex, caudate and putamen in UCO group compared to the control group by showing significantly lower number of branching intersections from the cell soma at distances of 20–120 μm (*p* < 0.05) (Figure 6). In the subiculum region, significant disturbance in the STT cells arborization in the UCO group was observed by showing a significant effect of UCO on the number of STT dendritic intersections at 40–100 μm from the soma compared to control (*p* < 0.001). In the EC, there were significant differences in STT dendritic complexity at 20–80 μm from the soma in UCO compared with the control group (*p* < 0.001) (Figure 6).

### Effect of UCO on the Oligodendrocyte Number

**FIGURE 7:**
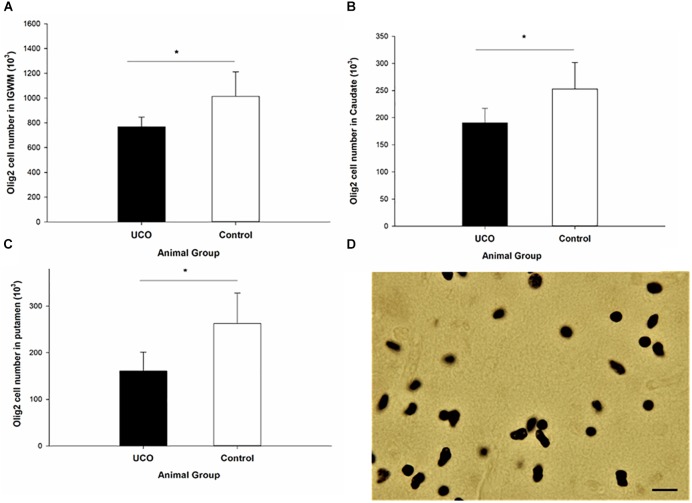
The number of Olig-2 cells was significantly lower in **(A)** IGWM, **(B)** caudate, and **(C)** putamen areas in UCO group compared to the control group. ^∗^*p* < 0.05. **(D)** Olig-2 positive cells with dark brown soma. Scale bar = 10 μm.

**FIGURE 8:**
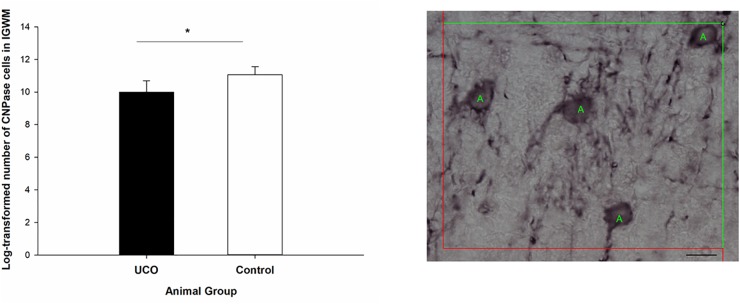
Log transformed number of CNPase positive cells in the IGWM area of the UCO and the control groups. IGWM, intragyral white matter. ^∗^*p* < 0.05 (left). Illustration of quantification of the number of CNPase cells in IGWM area of preterm fetal sheep (right). Scale bar = 10μm.

Umbilical cord occlusion was associated with a significant loss of Olig2 cells in IGWM, thalamus, putamen and caudate compared to control group (*p* < 0.05; Figure 7 and Supplementary Table 2), however, no significant difference in the number of Olig2 cells was observed in cortex and PVWM compared to the control group (*p* > 0.05). Counting the number of CNPase positive cells was performed in IGWM area and the Levene’s test showed significant difference in variance homogeneity and therefore a logarithmic transformation before applying independent *t*-test was done. There was a significantly reduced number of CNPase positive cells in UCO animals compared to the control group (*p* = 0.01) (Figure 8).

### Effect of UCO on the Neuronal Number

**FIGURE 9:**
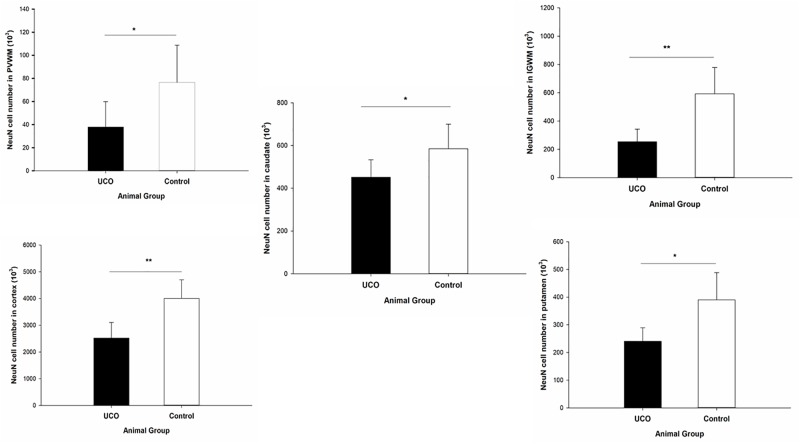
Effect of UCO on the number of NeuN positive neurons in IGWM, cortex, PVWM, putamen, and caudate. ^∗^*p* < 0.05, ^∗∗^*p* < 0.01.

Quantification of the number of mature (NeuN positive) neurons was done in cortex, IGWM, PVWM, putamen and caudate. The results indicated a significant negative effect of UCO on the number of NeuN positive cells in all these areas (*p* = 0.003; *p* = 0.004; *p* = 0.04; *p* = 0.01; *p* = 0.05) (Figure 9 and Supplementary Table 3).

## Discussion

To study the effect of prenatal asphyxia on the population of STT cells, we examined the number and morphology of STT cells in several brain regions 14 days following UCO in preterm fetal sheep. The animal model is well-established and regarded as suitable to examine the contribution of fetal asphyxia to neuropathological changes observed in infants born preterm (Koehler et al., 2018). Consistent with previous reports using the UCO model in preterm sheep, we found loss of NeuN positive neurons, particularly in subcortical gray matter areas, and reduced number of oligodendrocytes (Mallard et al., 2003; Bennet et al., 2007; van den Heuij et al., 2017). We provide new evidence demonstrating considerable dysmaturation of STT interneurons in different brain regions by showing a significant decrease in dendritic arborization of STT cells. While UCO resulted in a significant reduction in the number of STT interneurons in some brain regions, in the cerebral cortex, maturational delay occurred in the absence of loss of STT-positive cells. Further, while UCO resulted in a significant impairment of the morphology of STT cells in various brain regions, gross assessment of the brain injury did not concur with these alterations. Therefore, our results suggest high specific susceptibility of STT interneuron maturation to an asphyxial insult. Speculatively, this might impact on the balance between inhibitory and excitatory networks and could affect brain plasticity (Markram et al., 2004). A weakness of the study is that due to the size of the experimental groups, sex-dependent effects were not possible to determine. Further, the study would have been strengthened by addressing the role of neuroinflammation, such as effects on microglia and astrogliosis (GFAP).

About 20–30% of all neurons within the cerebral cortex are GABAergic interneurons and there are various subtypes of GABAergic interneurons, which have different spatial and temporal origins (Riedemann et al., 2016). GABAergic STT positive interneurons are the earliest born interneurons that migrate to the deep layers of the cortical plate during brain development (Miyoshi et al., 2007). It has been suggested that STT interneurons exhibit transient early synaptogenesis that is essential for the establishment of pavalbumin-dependent thalamocortical inhibition (Tuncdemir et al., 2016). A post-mortem study in infants born preterm (25–32 weeks gestation) showed that GABAergic interneurons are particularly vulnerable when also white matter injury is present (Liguz-Lecznar et al., 2016). It was also found that neuropeptide Y-positive interneurons had shorter neurite lengths and there was a significantly lower number of calretinin-positive cells in telencephalon in these infants, which was suggested to contribute to impairment of cortical development (Robinson et al., 2006). Another clinical study found significant lower parvalbumin-positive neuronal density in 10 of 11 patients with severe periventricular leukomalacia (PVL), which correlated with developmental impairment of thalamocortical connections (Iai and Takashima, 1999). In an experimental *in utero* injury model with cortical dysplasia in mice, significant reduction in the total number of neurons and GABAergic interneurons in the neocortex was evident in early post-natal life (Deukmedjian et al., 2004). A recent study showed that global cerebral ischemia in near term fetal sheep causes significant loss of GABAergic interneurons throughout the parasagittal cortex more specifically in cortical layer 6 (GAD+: by ∼88%; PV+: by ∼86%) at 1 week of recovery. It was suggested that the underlying mechanism of these changes was downregulation of interneuron markers on surviving neurons rather than cell death (Fowke et al., 2018). However, the previous studies did not examine the effect of perinatal brain damage on the number or maturation of the STT interneuron population. In the present study, we demonstrate that the number of STT interneurons is reduced in several subcortical regions including caudate, putamen, subiculum and ventral hippocampal DG, as well as EC, after UCO and the remaining STT neurons in these regions display altered morphological features. These regions are known to be sensitive to UCO resulting in neuronal loss as previously shown (Mallard et al., 2003; Bennet et al., 2007; van den Heuij et al., 2017). However, in the parasagittal cortex of UCO animals, morphological analysis of STT interneurons showed reduction of dendritic arborization and complexity of the dendritic branching pattern, which was not associated with an overall reduction in the number of STT positive cells. This finding indicates specifically perturbed maturation of STT cells rather than changes in interneuron genesis or apoptosis in parasagittal cortex following UCO. Interestingly, we could also detect a reduction of STT positive cells in the IGWM. These cells have been described in humans and primates as interstitial neurons that represent a specific population which is distinct from subplate neurons and neurons in adjacent structures (Judas et al., 2010). In fact, interstitial neurons may be future GABAergic neurons that tangentially migrate from the ganglionic eminence to the cortex in development (Yang et al., 2011). Overall our data draws the attention to maturational defects in the STT neuronal population after in utero asphyxia. Recently, two subtypes of STT interneurons, with distinct electrophysiological and morphological characteristics, were described (Riedemann et al., 2018). The neurochemical and functional diversity of STT interneurons may correlate with their vulnerability to utero asphyxia, which future studies should address.

It has been indicated that preterm birth results in activation of mechanisms such as dysynchrony of neurodevelopmental processing (Curristin et al., 2002) that can lead to maturational delay of both neurons and non-neuronal cells with the consequences of abnormality in circuit formation and function (Penn et al., 2016). Investigation of neuronal morphological development in premature infants suggests retardation of neuronal maturation (Takashima et al., 1982). In our study, the morphologic abnormalities in STT interneurons seen in different brain regions in the UCO group suggests delayed or arrested dendritic development of STT cells that may correspond to dysmaturation of cells. Vinall et al. (2013) demonstrated that the microstructural maturation rate of gray matter is lower in preterm infants in comparison with term infants due to multiple mechanisms including neuronal and non-neuronal cell death (Kinney et al., 2012) and disturbances in cellular arborization which may contribute to retarded maturation and underlie cognitive and learning disabilities in survivors of prenatal cerebral ischemia (Dean et al., 2013). Importantly, it has been shown that significant reduction of dendritic arborization and spine density in cortical and caudate projection neurons occurs without significant neuronal loss and therefore, it was concluded that reduction in dendritic arborization resulted from disrupted maturation, rather than from degeneration. In our study, we did find significant reduction in the number of NeuN positive cells in the cortex without significant changes in the number of STT cells. This finding may be related to the higher expression of hypoxia-inducible factor-1 (HIF-1) demonstrated in cortical interneurons following hypoxic-ischemic insults (Ramamoorthy and Shi, 2014).

It is known that the morphology of neuropeptide Y-positive neurons with regard to the average length of the longest neurite is significantly shorter in infants with perinatal brain injury and this may contribute to the predisposition to epilepsy in premature infants (Robinson et al., 2006). It has been shown that 30 min UCO in preterm sheep is associated with abnormal EEG epileptiform activity (George et al., 2004) and loss of GABAergic interneurons was shown to be associated with epilepsy in mice (Asada et al., 1996; Schuler et al., 2001). However, a recent study did not find a relationship between loss of interneurons and seizure burden following cerebral ischemia in the near term fetal sheep (Fowke et al., 2018). Instead it was suggested that degradation of perineuronal nets may lead to increased interneuron excitability and seizure-like activity (Vedunova et al., 2013; Fowke et al., 2018). Accumulating clinical and experimental evidence also suggest an important role of subiculum in epilepsy and epileptogenesis by indicating that physiology and distribution of neuronal and interneuronal cells in subiculum can contribute to the pattern of epileptiform firing (Stafstrom, 2005). According to this hypothesis, the reduction of STT interneurons with morphological disturbances in the subiculum of UCO animals observed in the present study may explain the seizure activity previously reported in this model (Iai and Takashima, 1999).

Our results indicated a significant reduction in the number of STT cells in the ventral hippocampal DG but not in the dorsal DG. Previous studies in rodents indicate that STT interneurons are more abundant with a higher percentage of total GABA neurons in the ventral than in the dorsal DG (Kosaka et al., 1988; Jinno and Kosaka, 2003). STT neurons are known to be vulnerable to ischemia (Johansen et al., 1987; Esclapez and Houser, 1995) and the DG is a brain region with high rate of neurogenesis. Therefore, significant alteration of interneuron genesis or apoptosis in the ventral hippocampal DG may have contributed to the significant decrease in the number of STT cells in this area following UCO.

Regarding the impact of UCO on the number of oligodendrocytes, we found fewer Olig-2 and CNPase positive cells in IGWM in the UCO group compared with control group. This is consistent with previous studies in preterm fetal sheep that have shown a decline in both of these cell populations following UCO (Mallard et al., 2003; Bennet et al., 2007), but in contrast to others that reported a reduction in CNPase positive cells in IGWM, PVWM, without a change in number of Olig-2 positive oligodendrocytes (Drury et al., 2014; van den Heuij et al., 2017). Disturbances of oligodendrocyte development is dependent on the timing and location (Marin-Padilla, 1997). In brains of infants with severe focal lesions in the white matter (PVL), there is a notably effect on the development of oligodendrocytes (Iida et al., 1995; Robinson et al., 2006) and studies have shown an increased Olig-2 cell population in proximity to necrotic white matter lesions (Billiards et al., 2008). A preserved Olig-2 cell density is believed to be due to robust proliferation of pre-OLs, but with subsequent failure to terminally differentiate, resulting in myelin deficiency (Back and Rosenberg, 2014). However, in our study, we found significant loss of both CNPase positive and Olig-2 positive oligodendrocytes in the white matter which may correspond to cell death as well as maturational arrest of oligodendrocytes.

## Conclusion

On the basis of our findings showing an abnormal maturation pattern in a population of GABAergic interneurons, STT interneurons, after UCO in preterm fetal sheep, we speculate that these changes could contribute to neurodevelopmental delay in preterm infants and impact on the balance between inhibitory and excitatory networks and may implicate a novel target for neuroprotective therapies.

## Data Availability

All datasets generated for this study are included in the manuscript and/or the Supplementary Files.

## Ethics Statement

Animal experiments were approved by the local Animal Ethics Committee of Gothenburg (No. 166/13) and performed according to the guidelines for animal experimentation by the Swedish Department of Agriculture.

## Author Contributions

CM, HH, AB, VS, and MA designed the study. MA, PS, AB, VS, and JE performed the experiments, data collection, and data analysis. CM and MA interpreted the results and wrote the manuscript. All authors provided the conceptual advice, commented on the manuscript, and approved the final version of the manuscript for submission.

## Conflict of Interest Statement

The authors declare that this study received funding from GlaxoSmithKline. The funder had some involvement in the study design, but no role in data collection and analysis, decision to publish, or preparation of the manuscript. All authors declare no conflict of interest.

## Footnotes

1 https://msu.edu/user/brains/brains/sheep/scans/0720/image1.html
