# Supplementary material for: Dysmaturation of Somatostatin Interneurons Following Umbilical Cord Occlusion in Preterm Fetal Sheep
Source: Front Physiol. 2019 May 22;10:563. doi: 10.3389/fphys.2019.00563 (PMC6538799; doi:10.3389/fphys.2019.00563)
Supplement: Supplementary file 1 [file Table_1.DOCX]

**Supplementary Table 1:** The effect of UCO on the total number of STT cells in brain regions from block B and block C.

| **Block** | **Type of cells** | **Brain region** | **Total number of cells in UCO group** | **Total number of cells in control group** | **UCO vs. Control** |
| --- | --- | --- | --- | --- | --- |
|  |  |  | **Mean ± SD** | **Mean ± SD** | ***p*** |
| **B** | **STT Cells** | PVWM | 6829 ± 2476 | 8870 ± 2330 | 0.17 |
|  |  | Cortex | 397992 ± 103032 | 594406 ± 224747 | 0.10 |
|  |  | Caudate | 43679 ± 14545 | 58531 ± 7672 | 0.043 |
|  |  | Putamen | 34330 ± 5223 | 59324 ± 8908 | 0.000 |
|  |  | IGWM | 94352 ± 22713 | 141509 ± 36558 | 0.030 |
| **C** | **STT Cells** | Dorsal hippocampus | 7692 ± 3727 | 10708 ± 3939 | 0.21 |
|  |  | Ventral hippocampus | 13169 ± 4240 | 21430 ± 7436 | 0.05 |
|  |  | Whole hippocampus | 20862 ± 7714 | 32138 ± 9122 | 0.049 |
|  |  | Entorhinal cortex | 149705 ± 18073 | 181925 ± 25406 | 0.036 |
|  |  | Subiculum | 30071 ± 15839 | 58737 ± 20411 | 0.026 |

**Supplementary Table 2:** The effect of UCO on the total number of Olig-2 cells in brain regions from block B and block C.

| **Block** | **Type of cells** | **Brain region** | **Total number of cells (10^3^) in UCO group** | **Total number of cells (10^3^) in control group** | **UCO vs. Control** |
| --- | --- | --- | --- | --- | --- |
|  |  |  | **Mean ± SD** | **Mean ± SD** | ***p*** |
| **B** | **Olig2 Cells** | PVWM | 513 ± 61 | 520 ± 86 | 0.87 |
|  |  | Cortex | 1058 ± 94 | 1150 ± 212 | 0.39 |
|  |  | Caudate | 190 ± 27 | 253 ± 49 | 0.02 |
|  |  | Putamen | 161 ± 40 | 263 ± 65 | 0.01 |
|  |  | IGWM | 769 ±77 | 1014 ± 198 | 0.017 |
| **C** | **Olig2 Cells** | Cortex | 1281 ± 248 | 1358 ± 331 | 0.65 |
|  |  | IGWM | 1873 ± 224 | 2306 ± 361 | 0.04 |
|  |  | Thalamus | 1345 ± 301 | 2066 ± 329 | 0.003 |
|  |  | PVWM | 596 ± 753 | 754 ± 640 | 0.71 |

**Supplementary Table 3:** The effect of UCO on the total number of NeuN cells in brain regions from block B.

| **Block** | **Type of cells** | **Brain region** | **Total number of cells (10^3^) in UCO group** | **Total number of cells (10^3^) in control group** | **UCO vs. Control** |
| --- | --- | --- | --- | --- | --- |
|  |  |  | **Mean ± SD** | **Mean ± SD** | ***p*** |
| **B** | **NeuN Cells** | PVWM | 38 ± 21 | 77 ± 32 | 0.049 |
|  |  | Cortex | 2521 ± 585 | 4003 ± 696 | 0.003 |
|  |  | Caudate | 451 ± 81 | 585 ± 115 | 0.05 |
|  |  | Putamen | 241± 49 | 390 ± 98 | 0.011 |
|  |  | IGWM | 253 ± 89 | 593 ± 187 | 0.004 |
